# Ssa1-targeted antibody prevents host invasion by *Candida albicans*

**DOI:** 10.3389/fmicb.2023.1182914

**Published:** 2023-07-25

**Authors:** Xi-Ran Qiu, Chen-Rui Shen, Li-Wen Jiang, Peng Ji, Yu Zhang, Wei-Tong Hou, Wen Zhang, Hui Shen, Mao-Mao An

**Affiliations:** ^1^Department of Pharmacology, Shanghai Tenth People’s Hospital, Tongji University School of Medicine, Shanghai, China; ^2^Department of Clinical Laboratory Medicine, Shanghai Tenth People's Hospital, Tongji University School of Medicine, Shanghai, China

**Keywords:** antibody, Ssa1, *Candida albicans*, systemic infection, antifungal

## Abstract

**Introduction:**

*Candida albicans* is a commensal fungus that colonizes most healthy individuals’ skin and mucosal surfaces but can also cause life-threatening invasive infections, particularly in immunocompromised patients. Despite antifungal treatment availability, drug resistance is increasing, and mortality rates remain unacceptably high. Heat shock protein Ssa1, a conserved member of the Hsp70 family in yeast, is a novel invasin that binds to host cell cadherins, induces host cell endocytosis, and enables *C. albicans* to cause maximal damage to host cells and induces disseminated and oropharyngeal disease.

**Result:**

Here we discovered a mouse monoclonal antibody (mAb 13F4) that targeting *C. albicans* Ssa1 with high affinity (EC_50_ = 39.78 ng/mL). mAb 13F4 prevented *C. albicans* from adhering to and invading human epithelial cells, displayed antifungal activity, and synergized with fluconazole in proof of concept *in vivo* studies. mAb 13F4 significantly prolonged the survival rate of the hematogenous disseminated candidiasis mice to 75%. We constructed a mAb 13F4 three-dimensional structure using homology modeling methods and found that the antigen-binding fragment (Fab) interacts with the Ssa1 N-terminus.

**Discussion:**

These results suggest that blocking Ssa1 cell surface function may effectively control invasive *C. albicans* infections and provide a potential new treatment strategy for invasive fungal infections.

## Introduction

*Candida albicans* can be an opportunistic pathogen (Witchley et al., 2019) that is delicately balanced with host immune recognition as part of the normal microbiota of the skin and mucous membranes (Kullberg and Arendrup, 2015; Netea et al., 2015; Stop neglecting fungi, 2017). When the local or systemic host defense mechanisms are compromised, *C. albicans* has the potential to undergo pathogenicity, causing diseases ranging from superficial mucosal infections (oral and vaginal) to invasive candidiasis involving bloodstream infection (candidemia) (Talapko et al., 2021). Invasive candidiasis represents a critical medical challenge intricately tied to the progress of medical technology, and it is extensively acknowledged as a prominent determinant of morbidity and mortality within the healthcare milieu (Magill et al., 2014; Cleveland et al., 2015; Kullberg and Arendrup, 2015; McCarty and Pappas, 2016). In hospitalized patients, *Candida spp.* takes the lead as the primary causative agent of fungal infections and holds the fourth position as one of the most frequent causes of nosocomial bloodstream infections (BSIs) (Fioriti et al., 2022). Regrettably, the efficacy of anticandidal therapy in these patients is limited, leading to an unacceptable high mortality rate of 50% (Kullberg and Arendrup, 2015; McCarty and Pappas, 2016). Disseminated candidiasis is particularly life-threatening in immunocompromised patients or has other exogenous factors (i.e., broad-spectrum antibiotic treatment, intravenous administration, transplantation medicine, trauma, or abdominal surgery). Due to the increasing number of immunocompromised patients and the aging population, we may see an increase in *C. albicans* infections in the years to come (Logan et al., 2020).

Apart from host immune defenses, there are also physical barriers between different tissues to prevent the spread of microorganisms (Lemichez et al., 2010; Bystrom et al., 2019). Invasive *C. albicans* infection involves several steps, including mucosal epithelium damage and invasion, vascular dissemination, yeast cell seeding into the bloodstream, and target tissue invasion and colonization. Damaging and invading epithelial or endothelial cells is critical for *C. albicans* to establish an invasive infection (Mba and Nweze, 2020). Therefore, interrupting this process is a potentially valuable method for preventing invasive candidiasis.

The cell wall of *C. albicans* has numerous important biological functions and serves as a significant source of fungal antigens (Gil-Bona et al., 2018; García-Carnero et al., 2020). Among cell wall proteins, heat shock proteins play essential roles in many organisms. For example, some heat shock proteins function as adhesins (Ratnakar et al., 1996; Long et al., 2003). Ssa1 is a member of the Hsp70 family in *C. albicans* and expresses on the yeast and hyphae cell wall surfaces (Li et al., 2003; Vylkova et al., 2006). Previous studies have indicated that Ssa1 is critically important for normal virulence in disseminated candidiasis and oropharyngeal candidiasis murine models. Additionally, the ssa 1Δ/Δ mutant exhibits reduced virulence in mouse models and has defects in binding to the N-cadherin and E-cadherin receptors, which mediate the host cell endocytosis of *C. albicans* (Sun et al., 2010). Other results suggest that Ssa1 is a potential drug target for invasive candidiasis (Weissman et al., 2020).

Only four antifungal drug classes are available for treating invasive candidiasis (Tragiannidis et al., 2013; Sanglard, 2016). Since echinocandins were discovered more than ten years ago, no new antifungal drug class has been introduced. Emerging resistance to these antifungal agents and toxicity necessitates alternative treatment strategies (Lee et al., 2021). Monoclonal antibodies (mAbs) have enormous potential as therapeutic agents and have made outstanding contributions to current oncology and autoimmune disease therapies (Chames et al., 2009; Buss et al., 2012; Ecker et al., 2015). Recent work has demonstrated that mAbs can be effective against microbes (Wu et al., 2020; Raj et al., 2021; Ai et al., 2022), and several studies have established that humoral immunity is potentially effective in host protection against *C. albicans*. Indeed, there is great interest in the possible benefits of mAb-based therapies for various fungal infections, including *Candida* infections (Cabezas et al., 2010; Sui et al., 2017; Rosario-Colon et al., 2021).

In this study, we developed a mouse monoclonal antibody (mAb 13F4) to address the aforementioned limitations and enhance the effectiveness of antifungal therapy. This study suggests the possibility of a novel therapeutic agent for *Candida albicans* infection, improving the efficacy of antifungal therapy while reducing the toxic side effects of small molecule drugs. By hybridoma screening, we obtained mAb 13F4, which showed good antifungal efficacy *in vitro* and *in vivo*.

## Materials and methods

### Ethics statement

All animal experiments were performed using procedures outlined by the Regulations on the Administration of Laboratory Animals approved by the State Council of the People’s Republic of China. The animal experiment protocol was verified and approved by the Animal Care and Use Committee of Tongji University (Permit Number: TJAA08021102).

### Mice

Female C57BL/6 mice and BALB/c mice were obtained from the Shanghai Laboratory Animal Center of the Chinese Academy of Sciences (Shanghai, China).

### Reagents, antibodies, and plasmids

IPTG (isopropyl-β-D-thiogalactopyranoside) and DTT (dithiothreitol) were purchased from Sangon Biotech. Ni-nitrilotriacetic acid (Ni-NTA) was purchased from QIAGEN. Fluconazole and DMEM medium were purchased from Sigma-Aldrich. FITC-labeled secondary antibody was purchased from Invitrogen. The LDH Cytotoxicity Assay Kit and normal Mouse IgG were obtained from Beyotime. PrimeSTAR^®^ Max DNA Polymerase was obtained from TaKaRa Bio. HRP-labeled goat anti-mouse antibody was purchased from Dingguochangsheng Biotechnology. pET-21a (+) was purchased from Novagen.

### *Candida* spp. growth conditions

All strains were maintained on sabouraud dextrose agar plates (1% peptone, 4% dextrose, and 1.8% agar) and grown in yeast peptone dextrose (YPD) medium (1% yeast extract, 2% peptone, and 2% dextrose) at 30°C for 12–14 h. *C. albicans* SC5314 was kindly provided by Sanglard D (Centre Hospitalier Universitaire Vaudois). Dr. Scott G. Filler generously gifted the ssa1Δ/Δ and ssa1Δ/Δ::SSA1 strains.

### *Candida albicans* Ssa1 protein cloning and expression

The gene encoding *C. albicans* SSA1 was cloned into pET-21a (+) with a 6 × His tag according to UniProtKB-P41797. The plasmids were then transformed into BL21 (DE3) pLysS cells for protein expression. The Ssa1 sequences were confirmed by automated DNA sequencing. Ssa1 was expressed in the BL21 *E. coli* strain. A 2 mL overnight culture was grown in LB plus ampicillin, then diluted 1:100 in a 100 mL culture and grown at 37°C to an OD_600_ of 0.6–0.8. IPTG (Sangon Biotech) was added to the culture to achieve a final concentration of 0.1 mM, and the culture was incubated for an additional 16 h at 16°C. Cells were harvested by centrifugation and stored at −80°C.

### Purification of rSsa1-His

Bacterial cell pellets were thawed on ice and resuspended in sterile PBS. Cells were lysed in an ultrasonic cell disruption system, and the crude lysate was clarified by centrifugation at 13000 rpm for 30 min at 4°C. Following 0.2 μm filtration, the supernatant was loaded onto a 2 mL Ni-NTA-Agarose column (Qiagen) equilibrated with Lysis buffer (containing 20 mM imidazole). Recombinant His-tagged Ssa1 (rSsa1-His) was purified with Washing buffer (containing 100 mM imidazole) and eluted with 10 mL elution buffer (containing 250 mM imidazole). The 10 mL fractions containing rSsa1-His were dialyzed into 1 × PBS (pH 7.2), and aliquots were frozen at −80°C.

### Immunization and hybridoma generation

Eight-week-old BALB/c mice received three rounds of subcutaneous rSsa1-His injections at multiple sites following the repetitive immunizations multiple sites (RIMMS) immunization protocol (Kilpatrick et al., 1997). Mice were immunized over 28 days at 14 days intervals. For each immunization round, 0.5 mg/mL or 0.25 mg/mL rSsa1-His was emulsified in complete Freund’s adjuvant or incomplete Freund’s adjuvant (Sigma) and injected at the enterocoelia, groin, dorsum, and root of the tail. The volume of protein and adjuvant was 1:1. Test bleeds were collected on day 35 and assayed using an antigen enzyme-linked immunosorbent assay (ELISA). On day 42, mice were given rSsa1-His intravenously. Mice were maintained in SPF grade environments throughout the immunization cycle. Splenocytes were fused with myeloma (P3X63Ag8.653) to generate stable hybridomas (Köhler and Milstein, 1975).

### Chimeric anti-Ssa1 mAb expression and purification

Clarified murine hybridoma supernatants were concentrated by centrifuge at 4°C. The concentrated supernatants were then incubated with 2 mL protein G for 2 h, and the bound IgG was eluted with 0.1 M glycine (pH 2.5) and neutralized to pH 9.0 with 1 M Tris–HCl. The neutralized material was replaced and concentrated using a 50 kDa ultrafiltration device (Millipore). The IgG-containing buffer was then replaced with PBS (pH 7.2).

### Enzyme-linked immunosorbent assay

Ninety-six-well plates (Thermo Scientific, 442404) were coated with recombinant *C. albicans* Ssa1 or the indicated *C. albicans* Ssa1 domains (0.1 μg/well) 4°C overnight and then blocked with 200 μL PBST (PBS containing 0.05% Tween-20) containing 5% bovine serum albumin (BSA). The flat bottom plates were then incubated 4°C overnight with serially diluted mAb 13F4 antibody. When used for hybridoma screening, 100 μL of cell supernatant of different clones is added to each well. When used for testing the affinity of mAb 13F4, the concentration of antibodies was 10 μg/mL in the first well. After incubation with 1:10000 dilutions of HRP-labeled goat anti-mouse antibody (Dingguo-Changsheng Biotechnology) for 1 h, 100 μL TMB (3,3′,5,5′-tetramethylbenzidine) substrate was added, and plates were incubated for 10 min. The 50 μL 2 M HCl was then added to stop the reaction. Finally, the reaction mixture absorbance was measured at 450 nm using a multi-mode microplate reader. A 96-well microplate coated with BSA was used as a parallel control to analyze plasminogen binding to recombinant *C. albicans* proteins. The parallel control data was used to determine non-specific antibody binding to the microplate.

### *Candida albicans* binding assay

Ninety-six-well plates (Thermo Scientific, 442404) were coated with *C. albicans* ssa1Δ/Δ or ssa1Δ/Δ::SSA1 strains (~10^7^ CFU/well) overnight and then blocked with PBST (PBS containing 0.05% Tween-20) containing 5% bovine serum albumin (BSA). The plates were then incubated with serially diluted mAb 13F4 at 37°C for 1 h. After incubation with an HRP-labelled goat anti-human antibody (Thermo Fisher) for 1 h, TMB (3,3′,5,5′-tetramethylbenzidine) substrate was added and plates were incubated for 15 min. 2 M HCl was then added to stop the reaction. Finally, the reaction mixture absorbance was measured at 450 nm using a multi-mode microplate reader.

### Flow cytometry comparison of anti-Ssa1 monoclonal antibody binding

Exponentially growing *C. albicans* SC5314 cells were incubated with different concentrations of mAb 13F4 at 4°C overnight. The *C. albicans* SC5314 cells (~10^6^ CFU) were then washed with PBS and incubated with 1:2000 dilutions of FITC-labeled secondary antibody (Invitrogen) at 30°C for 1 h. The stained *C. albicans* SC5314 cells were fixed with 1% formaldehyde overnight and analyzed by flow cytometry (BD FACSVerse).

### Confocal laser scanning microscopy

To stain the cell wall in order to locate the fungus, exponentially growing *C. albicans* SC5314 cells (~10^7^ CFU/mL) were incubated with mAb 13F4 (100 μg/mL), BSA (as a negative group) or PBS (as a model group) at 37°C for 30 min. The cells were then incubated in the dark with 1 μg/mL calcofluor white (CFW) for chitin for 10 min. FaDu cells were grown to an approximately 80%–90% confluent monolayer in DMEM with 10% (vol/vol) heat-inactivated fetal bovine serum (FBS). Each dish of FaDu cells was infected with 2 × 10^6^ live *C. albicans* yeast cells for 90 min (MOI = 5). Then the FaDu cells were incubated with 20 μg/mL PSA-FITC (Sigma) for 10 min in the dark. The stained cells were analyzed at 63× magnification with a confocal laser scanning microscope (LSM880). Micrographs were then acquired and analyzed by ZEN.

### *Candida albicans* adhesion assay

FaDu cells and Caco-2 intestinal epithelial cells were cultured in DMEM with 10% (vol/vol) heat-inactivated FBS at 37°C with 5% CO_2_. 1 × 10^6^ cells were co-cultured with 200 *C. albicans* SC5314 cells in the exponential growth phase in six-well plates for 1.5 h. The *C. albicans* SC5314 were incubated with mAb 13F4, BSA (as a negative group), or PBS (as a model group) at 37°C for 1 h before being added into the FaDu or Caco-2 cells. Afterward, non-adherent *C. albicans* were removed using sterile PBS. Subsequently, YPD agar was added into each well, and the plates were incubated at 30°C. After a 48 h incubation, colonies were counted as adhesive *C. albicans*. The average number of colonies was 108 for the mAb 13F4 group and 145 for the negative group.

### Host cell damage assay

KB and FaDu epithelial cells were cultured with DMEM containing 10% (vol/vol) heat-inactivated FBS in 96-well plates (2.5 × 10^4^ cells per well) at 37°C with 5% CO_2_. Then *C. albicans* (2.5 × 10^3^ cells per well) and mAb 13F4 or BSA were added to the 96-well plates. The *C. albicans* were incubated with mAb 13F4, BSA (as a negative group), or PBS (as a model group) at 37°C for 1 h before infecting the KB or FaDu cells. After co-culturing for 24 h, the cell supernatant was collected for an LDH assay using a commercially available kit (Beyotime, Cat#: C0016). Maximal LDH release was determined by adding an LDH releasing agent in parallel. Relative LDH release was determined as follows: LDH (%) = (OD_490_ indicated cells − OD_490_ control)/ (OD_490_ maximal LDH release − OD_490_ control).

### Murine systemic candidiasis model

To study *in vivo C. albicans* infection, 6–8 weeks-old C57BL/6 female mice were intravenously injected with 200 μL of PBS containing live *C. albicans* SC5314 (1 × 10^6^ cells per mouse). mAb 13F4 was intravenously injected 2 h before infection at a dose of 20 mg/kg. Fluconazole was intraperitoneally injected 2 h after infection at 0.15 mg/kg. As for the model group, an unrelated antibody was intravenously injected 2 h before infection at a 20 mg/kg dose. Survival was then monitored for 12 days. The kidneys were removed 2 days post-infection and then homogenized in PBS buffer to determine fungal burden or fixed in 10% neutral formalin for hematoxylin-eosin (H&E) staining or periodic acid-Schiff (PAS) staining.

### Antigen-antibody homology modeling and docking

The antibody amino acid sequence was entered into the server,^1^ and homology modeling was performed based on the antibody sequence. The amino acid sequence of Ssa1 (Entry ID: P41797) of *Candida albicans* heat shock protein SSA1 was selected from the Uniprot database and homology modeling as described above. Upload the homologous modeling and optimized antibody and antigen structures to the Zdock server^2^ for protein–protein docking using the above method to obtain the optimal Pose (docking conformation).

### Statistical analysis

All experiments were performed on biological replicates, and the sample size for each experimental group per condition is reported in the appropriate figure legends and methods. A log-rank test was used to evaluate survival curve equality. The two-tailed student’s *t*-test was used for analyses of two groups, and multiple groups were analyzed by one-way ANOVA with Bonferroni’s *post hoc* comparisons. For analysis of nonparametrically distributed data, the Mann–Whitney test or Kruskal–Wallis test was used. *p*-values indicating statistical significance are indicated in the figures as: ^*^p < 0.05, ^**^p < 0.01, and ^***^p < 0.001.

## Results

### Preparation of high purity recombinant *Candida albicans* Ssa1 protein

Recombinant *C. albicans* Ssa1(rSsa1-His) protein was purified using Ni-NTA (Ni-nitrilotriacetic acid) under low imidazole conditions, eluted under high imidazole conditions to avoid any permanent structural changes, and checked for purity using SDS-PAGE (SDS- Polyacrylamide gel electrophoresis). Ssa1 (UniProtKB: P41797) in *C. albicans* SC5314 is ~70 kDa and has ATPase activity. Our data indicate that the purified protein has a specific band at ~70kda with a purity of approximately 90% (Supplementary Figure S1A) and ATPase activity (Supplementary Figure S1B), indicating that the rSsa1-His had a neutral structure.

### Ssa1-specific mAb screening, identification, and expression

**Figure 1:**
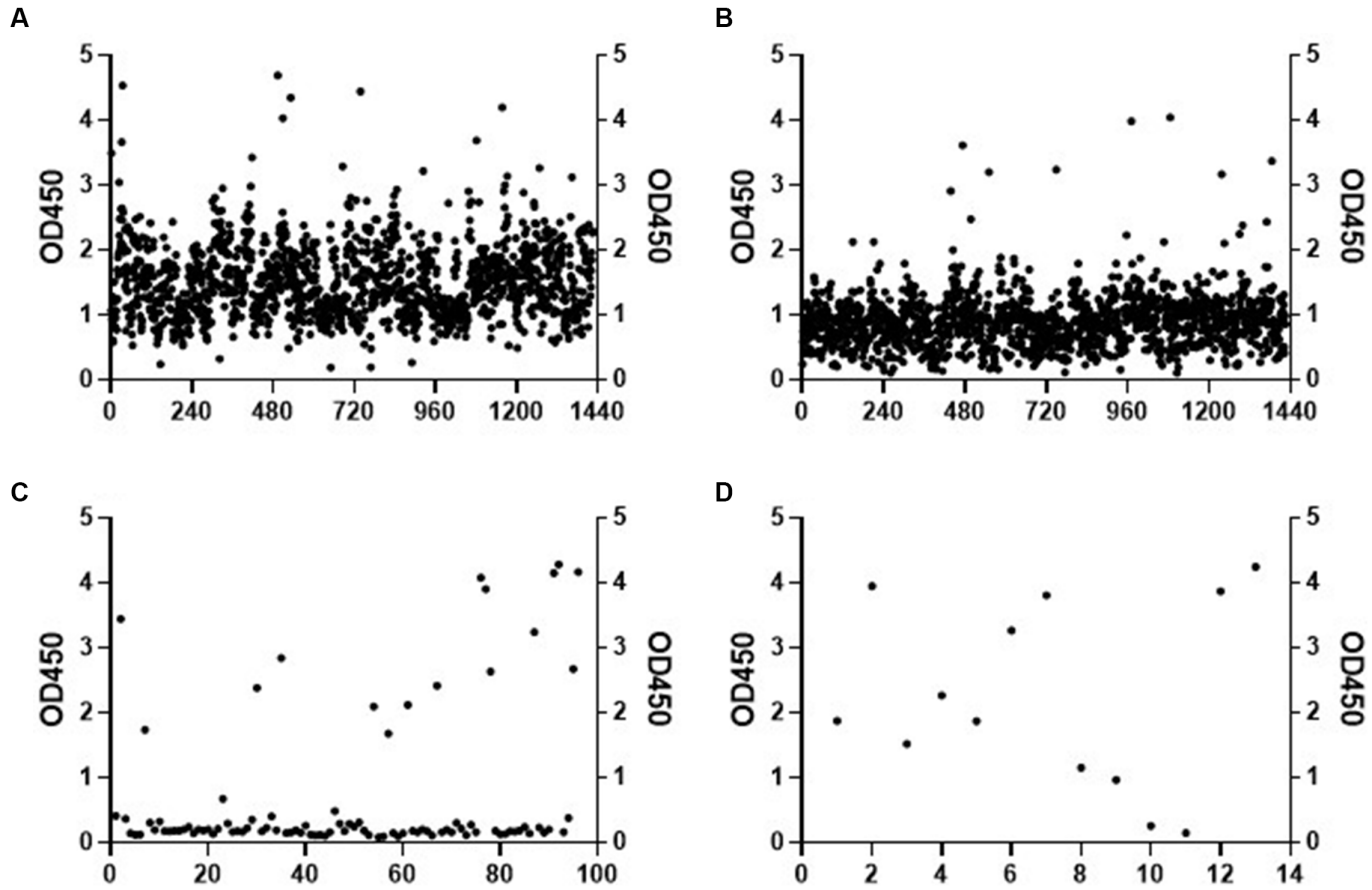
The screening of hybridoma cell line. The affinity of cell supernatant with Ssa1 was tested by ELISA (part of the experimental data). The positive clones in the first **(A)** and the second **(B)** screening were selected and amplified. After twice screenings **(C**,**D)** of the cell supernatant in the 24-well plate, the positive clones are selected for subcloning.

**Figure 2:**
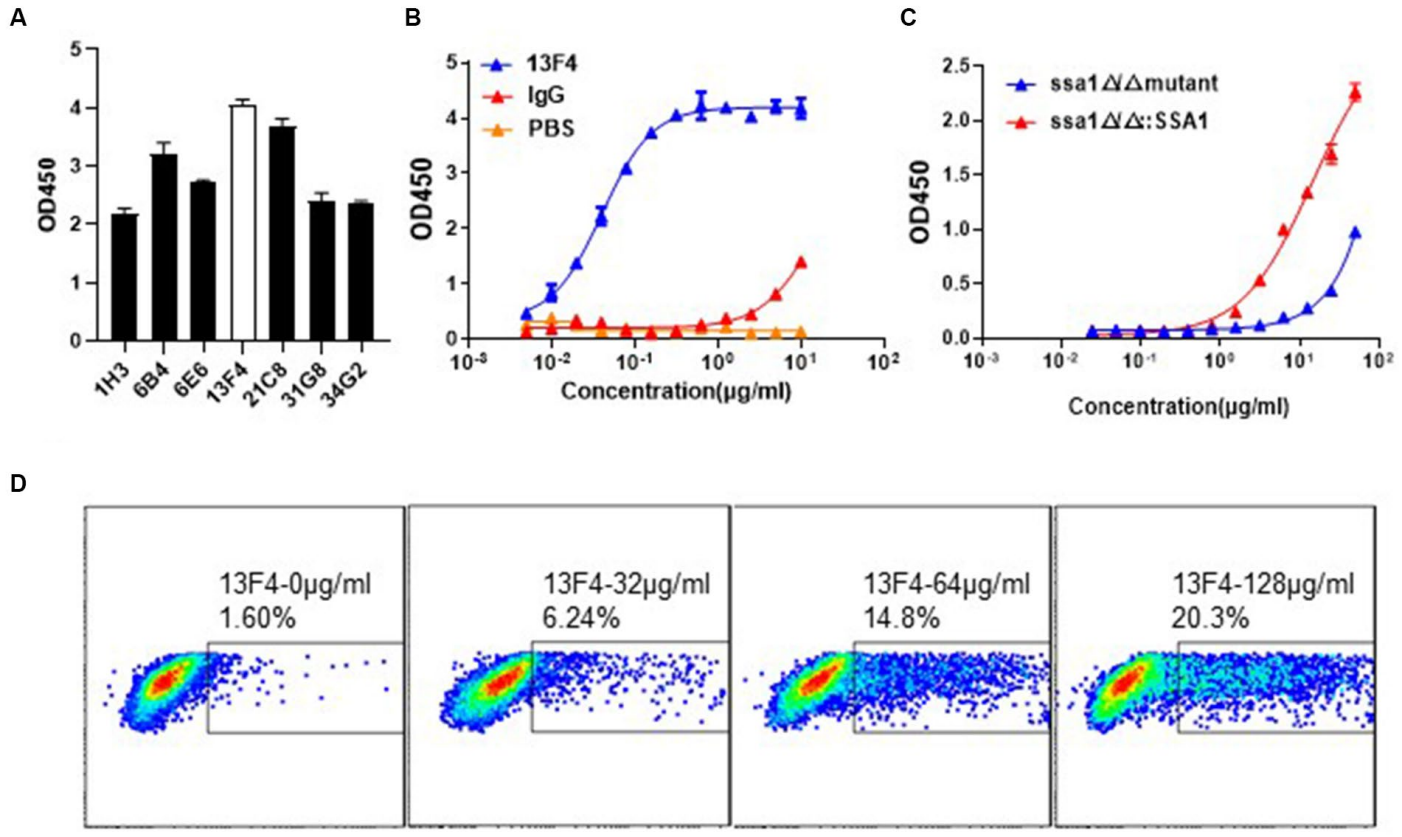
The binding assay of mAb 13F4 targeting *C. albicans* Ssa1. **(A)** The binding affinity of cell supernatant with Ssa1 was tested by ELISA. The mAb 13F4 is the top clone. **(B)** The cell supernatant of mAb 13F4 was purified and tested by ELISA. The concentration of antibodies is 10 μg/mL in the first well. Coating 0.1 μg/well Ssa1 protein in 96-well plate. EC_50_ = 39.78 ng/mL. **(C)** The binding affinity of mAb 13F4 with *C. albicans* ssa1 Δ/Δ and ssa1Δ/Δ::SSA1 strains. The concentration of antibodies is 50 μg/mL in the first well. Coating ~10^7^ CFU *C. albicans* in 96-well plate. mAb 13F4 could specifically recognize the Ssa1 on the cell wall surface of *C. albicans*. EC_50_ = 14.40 ug/mL. **(D)** *C. albicans* were incubated with mAb 13F4 of 128 μg/mL, 64 μg/mL, 32 μg/mL or 0 μg/mL, respectively. The samples were performed using a BD FACSVerse and analysis was performed using FlowJo analysis software.

To construct a mAb library, mice were immunized with recombinant *C. albicans* Ssa1 protein. After the RIMMS (repetitive immunizations multiple sites) immunization protocol, the polyclonal antibodies in serum were detected using ELISA. We observed high antiserum response levels after the third immunization (data not shown). Four thousand nine hundred ninety-two clones were screened in 96-well plates over two cycles for cell supernatant affinity, and 216 positive clones were developed (Figures 1A,B). We defined that the affinity between the cell supernatant of positive clones and rSsa1-His protein is higher than 1.5 (presented on OD_450_). We amplified these positive clones in 24-well plates and performed two additional screening cycles (Figures 1C,D). The top 7 clones were selected and subcloned by limiting dilution (Figure 2A). By screening the cell supernatant, we further obtained hybridoma cells that may be monoclonal. After sequence analysis of these positive clones, the best clone, which had the highest affinity of rSsa1-His (tested by ELISA), was identified (13F4) and shown to be a monoclonal antibody (mAb). mAb 13F4 was then expressed in hybridoma and purified using protein G. Our experimental results showed that mAb 13F4 had a high affinity for rSsa1-His (Figure 2B).

### mAb 13F4 recognizes natural Ssa1 protein on the *Candida albicans* surface

Microbe and host cells, including phagocytic cells, contact each other at the cell surface (Filler, 2006; Hooper et al., 2012). Cell wall proteins maintain structural integrity and mediate adherence, either to host cells or microbes and are potential antibody response targets. We performed flow cytometry analysis to determine if mAb 13F4 recognized natural *C. albicans* Ssa1 *in vitro*. To prove the assumption that mAb 13F4 only binds to Ssa1, we verified its binding affinity to the ssa1Δ/Δ or ssa1Δ/Δ::SSA1 strains. We found that mAb 13F4 could only recognize the ssa1Δ/Δ::SSA1 strain rather than the ssa1Δ/Δ strain, which indicated the specific binding affinity for Ssa1 (Figure 2C). Furthermore, we found that mAb 13F4’s specific binding to *C. albicans* increased in a dose-dependent manner, with 1.60% bound at 0 μg/mL, 6.24% bound at 32 μg/mL, 14.8% bound at 64 μg/mL, and 20.3% bound at 128 μg/mL (Figure 2D).

### Inhibiting Ssa1 protein exposure affects *Candida albicans* adhesive and invasive capacity

To persist and cause disease in the human host, *Candida albicans* must adhere to and invade host cells or tissues (Gow et al., 2011; Sheppard and Filler, 2014). In other organisms, heat shock proteins are essential to these functions. For instance, certain heat shock proteins expressed on the surface of microbial cells function as adhesins, facilitating adherence (Ratnakar et al., 1996; Hartmann and Lingwood, 1997). Furthermore, members of the Hsp70 and Hsp100 heat shock protein families play an indispensable role in resisting host-induced stress in certain bacteria and parasites (Hübel et al., 1997; Folgueira et al., 2008; Meibom et al., 2008). Ssa1 is highly expressed on the surface of yeast and hyphal cells, and it is one of the members of the Hsp70 family in *Candida albicans* (Meibom et al., 2008).

**Figure 3:**
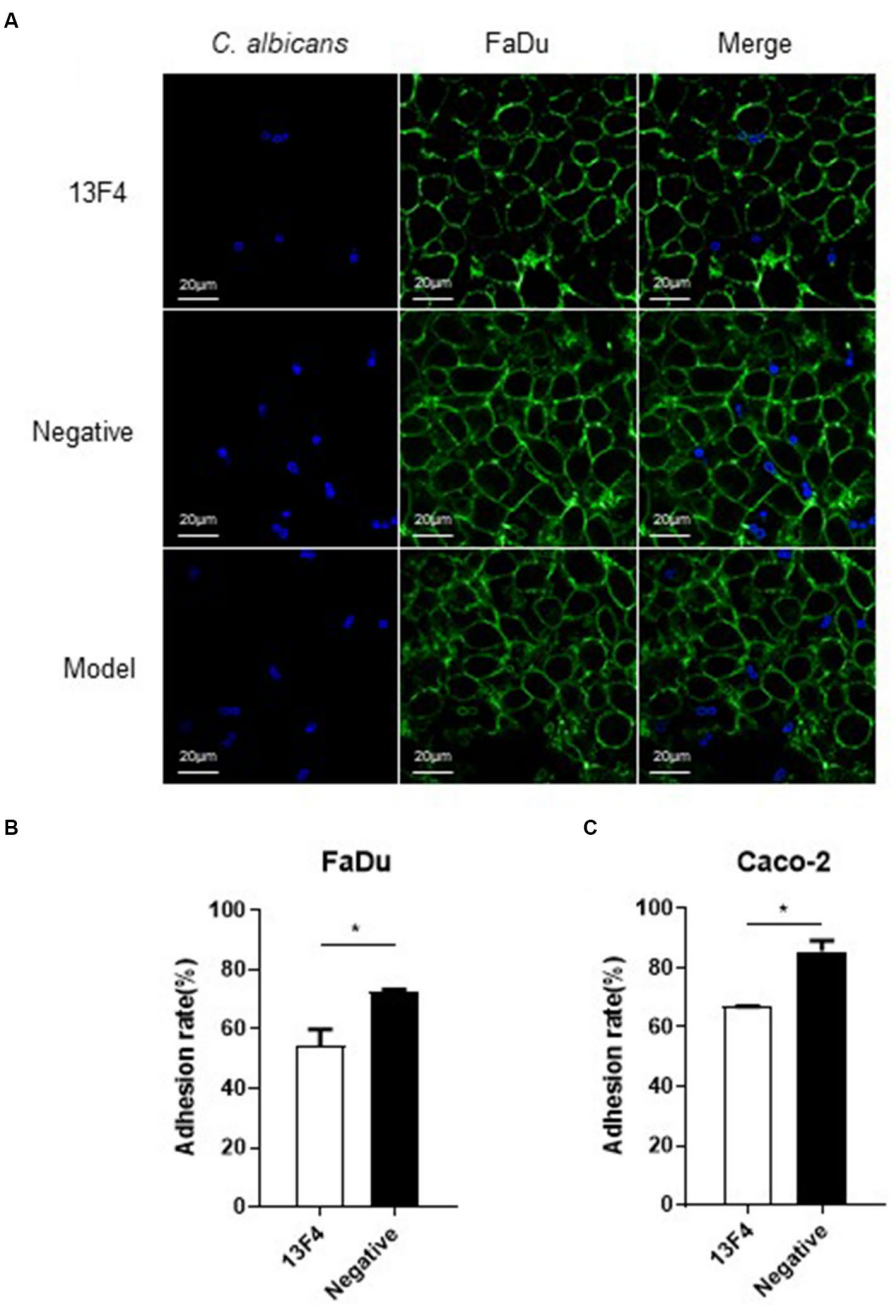
Blocking Ssa1 in cell wall can reduce the adhesion and endocytosis of epithelial cells by *C. albicans*. **(A)** Adhesion and endocytosis of FaDu cells by *C. albicans*. Live *C. albicans* was co-cultured with the FaDu cells grown on confocal dish for 90 min. After staining with CFW (1 μg/mL) and PSA-FITC (20 μg/mL) for 10 min, the samples were viewed by confocal laser scanning microscope directly. Scale bar represents 20 μm. Fluorescein isothiocyanate-conjugated *Pisum sativum* agglutinin (PSA-FITC), calcofluor white (CFW) and overlay are shown individually. **(B,C)** *C. albicans* SC5314 interacted with FaDu cells and Caco-2 cells, respectively, to observe the adhesion between the fungus and different epithelial cells. The experiment was repeated twice, and the data was expressed by mean ± SD. One-way ANONA test was used to analyze the statistical significance of the difference in adhesion rate between the groups. ^*^p < 0.05.

Moreover, Ssa1 is essential for *C. albicans* to invade endothelial cells and oral epithelial cells *in vitro* (Phan et al., 2013). Thus, we next investigated *C. albicans* SC5314 virulence while co-incubating with mAb 13F4 *in vitro*. The pharynx is a common site where *Candida albicans* invade the host, so we chose the FaDu cell (human pharyngeal squamous cell carcinoma cells) to test the function of mAb 13F4 *in vitro*. We used an interaction model to find that mAb 13F4 significantly reduced the *C. albicans* quantity in/on the epithelial cells compared to the negative control group. These results suggested that mAb 13F4 attenuated (~50%) *C. albicans* adhesion to and endocytosis of FaDu cells (Figure 3A). We observed three representative fields in each group. There were approximately 80 FaDu cells in each field.

Regarding the quantity of *C. albicans* endocytosed by FaDu cells, the model group had 17, the negative group had 15, and the 13F4 group had 6 (as shown in Figure 3A). Similar results were obtained for the other two representative fields in each group. Based on the adhesion assay methods described above, after co-culturing for 1.5 h, we observed that mAb 13F4 could significantly attenuate the adhesion between *C. albicans* and different types of epithelial cells *in vitro*, such as FaDu cells (Figure 3B) and Caco-2 cells (Figure 3C).

**Figure 4:**
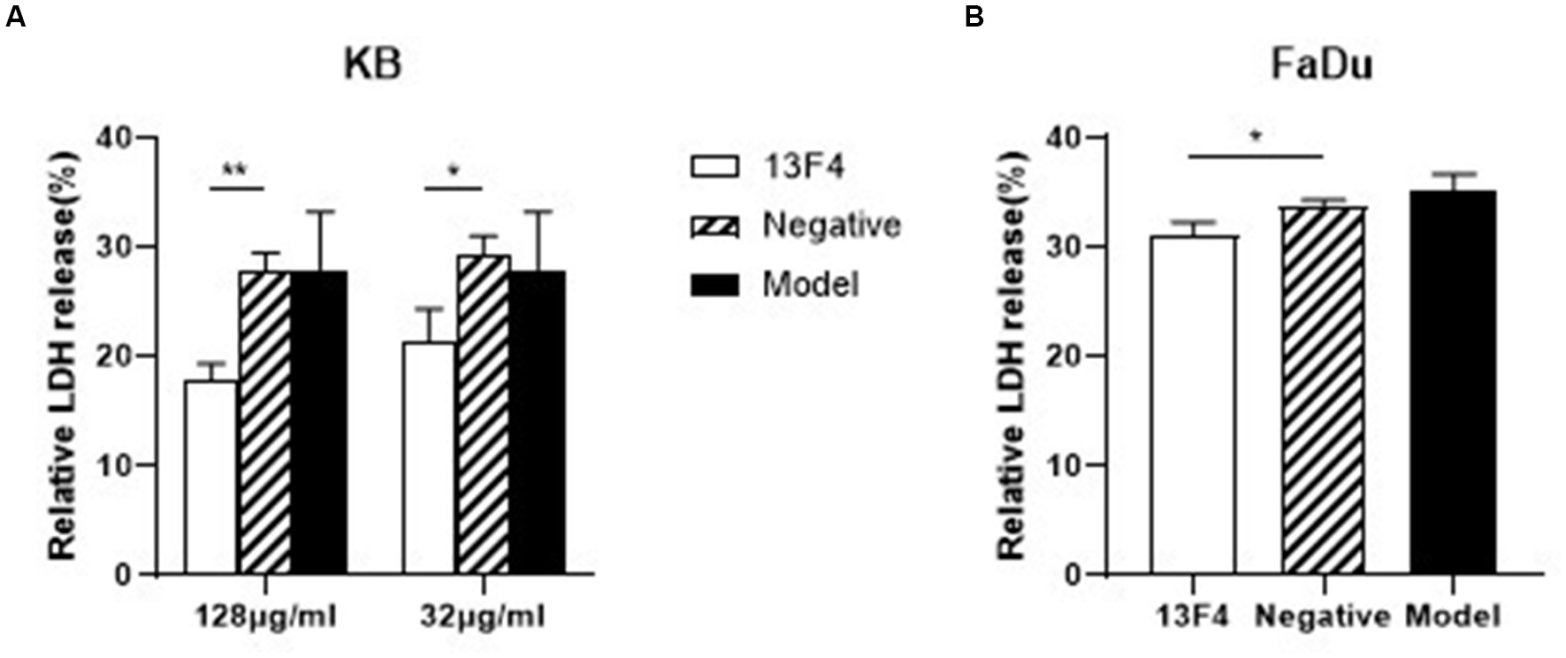
Blocking Ssa1 in cell wall can reduce the damage of host epithelial cells by *C. albicans*. *C. albicans* SC5314 interacted with KB cells **(A)** and FaDu cells **(B)** respectively, and the amount of LDH released in the supernatant was measured to assess the degree of damage to epithelial cells by *C. albicans*. mAb 13F4 could significantly inhibited the damage of KB cells by *C. albicans*. The experiment was repeated three times, and the data was expressed by mean ± SD. One-way ANONA test was used to analyze the statistical significance of the difference in LDH release rate between the groups. LDH, lactate dehydrogenase. ^**^p < 0.01, ^*^p < 0.05.

To develop an invasive infection, epithelial or endothelial cell damage is necessary for *C. albicans* (Kumamoto and Vinces, 2005; Gow et al., 2011). Therefore, to investigate whether mAb 13F4 could inhibit *C. albicans*-induced infection, we first tested mAb 13F4’s impact on *C. albicans*-induced damage to KB cells (Human oral epidermoid carcinoma cells) and FaDu cells (human pharyngeal squamous cell carcinoma cells), which are the possible lesion sites. After co-culturing for 24 h, we observed that KB cell lactate dehydrogenase (LDH) released into the supernatant significantly decreased with mAb 13F4 treatment. LDH release was suppressed by 9.8% compared to the negative group (Figure 4A). We also observed this effect in FaDu cells (Figure 4B). These data suggest that mAb 13F4 inhibited Ssa1 and attenuated *C. albicans* invasiveness *in vitro*.

### Anti-Ssa1 mAbs reduce hematogenous disseminated candidiasis in mice

**Figure 5:**
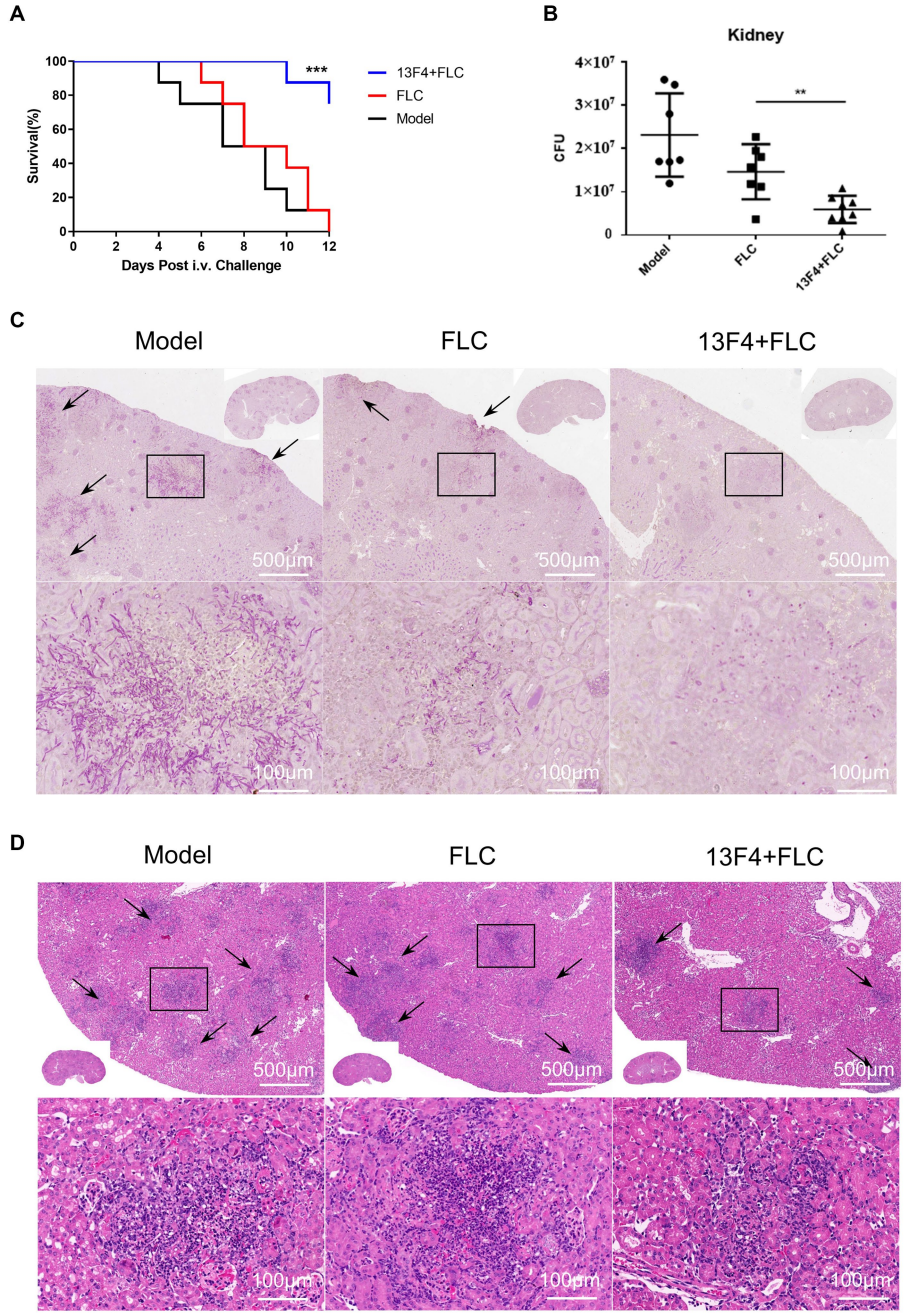
Blocking of Ssa1 is required for the reduction of *C. albicans* systemic infection. C57BL/6 mice were infected with 1 × 10^6^ CFU SC5314 in 200 μL sterile PBS via lateral tail vein. **(A)** Survival of C57BL/6 mice infected with the indicated strains was monitored for 12 days (*n* = 8 per group). A log-rank test was used to evaluate survival curve equality. Data are representative of three independent experiments. **(B)** Quantification of the fungal burden in kidney tissues of C57BL/6 mice (*n* = 8 per group) infected with indicated *C. albicans* at day 2. Data are representative of three independent experiments. Horizontal lines represent mean values, and whiskers representing standard deviations. ^**^p < 0.01 versus the result for the control (one way analysis of variance). **(C,D)** Representative PAS (for *C. albicans*) and H&E (for the inflammatory cells influx and the extent of tissue necrosis) staining of kidneys from C57BL/6 mice infected with indicated strains at day 2. Arrows indicate *C. albicans* filaments in the tissues. ^***^p < 0.001, ^**^p < 0.01.

Ssa1 is required for maximal virulence in a mouse model of hematogenous disseminated candidiasis (Sun et al., 2010). To investigate mAb 13F4’s therapeutic effects on *C. albicans* infection, we compared different therapeutic regimens in a hematogenous disseminated candidiasis mouse model. After treatment with mAb 13F4 and fluconazole, the survival rate was significantly higher than mice in the fluconazole or model group. Over a 12 days observation period, only two of the mice treated with mAb 13F4 and fluconazole died. By contrast, all mice in the model and fluconazole groups died within 12 days (*p* < 0.001, Figure 5A). Two days post-infection, mice treated with mAb 13F4 and fluconazole had significantly lower fungal burdens in the kidneys compared to those treated with either fluconazole or PBS (phosphate buffered saline) (*p* < 0.01, Figure 5B). Moreover, periodic acid-Schiff (PAS) staining identified more hyphae in the kidneys of fluconazole- or PBS-treated mice compared to mice treated with mAb 13F4 and fluconazole (Figure 5C). Additionally, hematoxylin and eosin (H&E) staining revealed that kidney inflammatory influx and tissue necrosis during infections were attenuated in mice treated with mAb 13F4 and fluconazole (Figure 5D). These results suggest that mAb 13F4 significantly relieved hematogenous disseminated candidiasis symptoms by binding Ssa1 *in vivo*.

### Ssa1·mAb 13F4 Fab complex structural analysis

**Figure 6:**
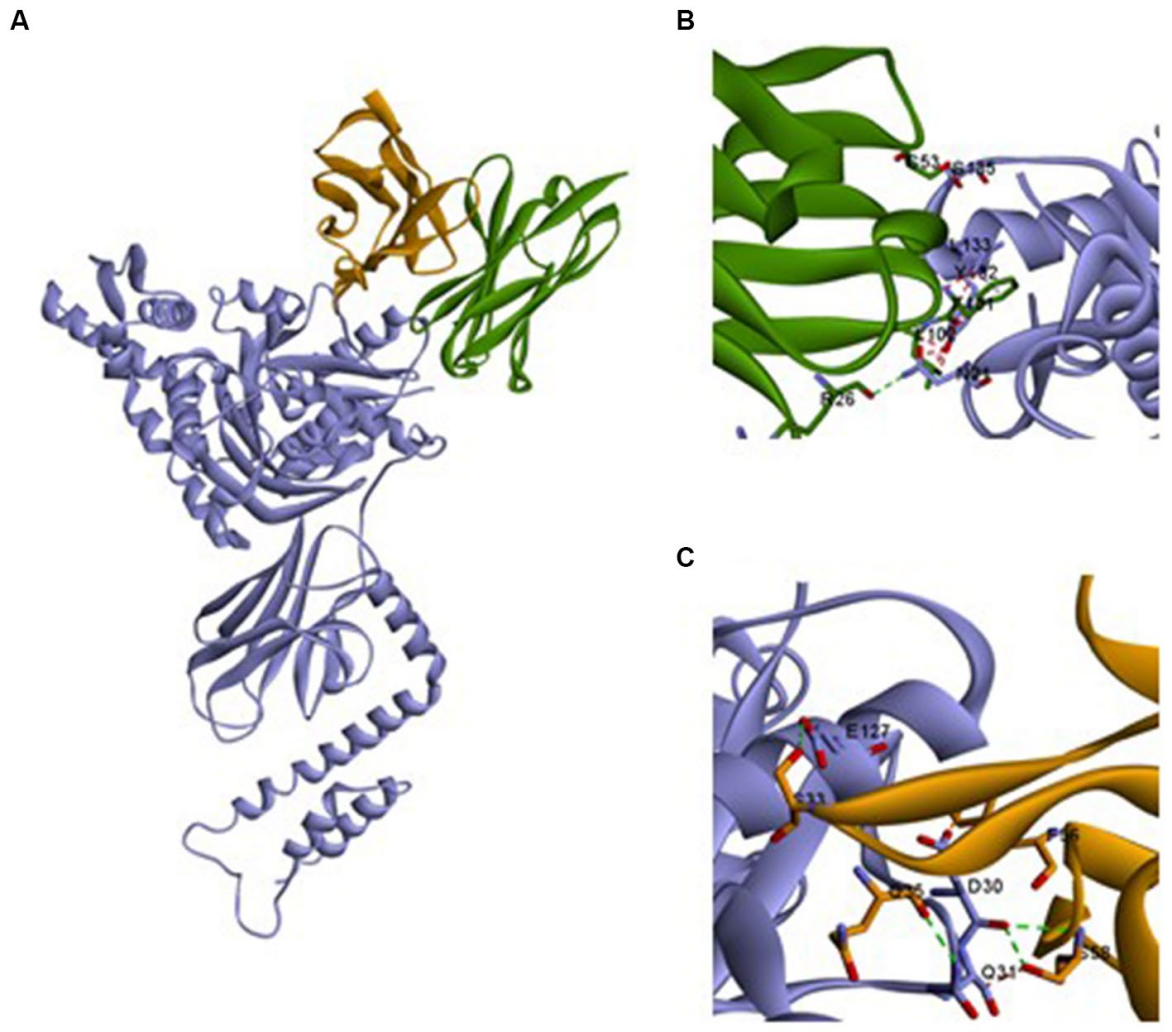
The structure of Ssa1-mAb 13F4 Fab complex. **(A)** Three-dimensional structure of the complex between monomeric Ssa1 (purple) and mAb 13F4 Fab [HC (green)/LC (orange)]. Interface between mAb 13F4 HC (green) and Ssa1 (purple) **(B)** and mAb 13F4 LC (orange) and Ssa1 (purple) **(C)**. Both chains of the Fab interact with Ssa1 and create hydrogen bonds (dotted lines). Residue Y132, L133 interact with heavy chain (HC) and D30 interacts with light chain (LC) bу *π*–*π* stacking interaction.

We first established the modelings to predict the interactions between Ssa1 and mAb 13F4 (see footnote 1). Then, the Ssa1·mAb 13F4 Fab complex were docked by https://zdock.umassmed.edu/. We examined the Ssa1·mAb 13F4 Fab complex structure and found that mAb 13F4 Fab binds the Ssa1 N-terminus at a non-linear epitope (Figure 6A). In the crevice, the Ssa1 molecule binds between the mAb 13F4 Fab light chain (LC) and heavy chain (HC). Residues TYR132-LEU133 interact with HC PHE101-LEU102 via a *π*–*π* stacking interaction, and residues ASN21 and SER135 interact with ARG26 and SER53 through hydrogen bonds (Figure 6B). Like the HC, residue ASP30 interacts with LC PHE56 and ASP30-GLN31, and GLU127 interacts with SER33, GLN35, and SER58 through hydrogen bonds (Figure 6C). Several hydrogen bonds are established between Ssa1 and mAb 13F4 Fab CDRH2 and between Ssa1 and mAb 13F4 Fab CDRL2.

## Discussion

Invasive candidiasis is a major fungal infection in humans, which can cause life-threatening diseases in immunocompromised patients and those with neutropenia, including patients in the intensive care unit (ICU) (Bugli et al., 2013; Wang, 2015). However, despite advancements in the development of drugs for invasive candidiasis, the incidence and mortality rates remain high. Generally, cellular immunity and innate immunity are considered the most crucial defense mechanisms against candidiasis (Fidel and Sobel, 1994; Naglik et al., 2017).

Antibody therapy is being increasingly applied to a wider range of human diseases, including metabolic diseases, hematological disorders and infectious diseases. The monoclonal antibody has strong specificity and high titer. It is mainly used for the diagnosis and treatment of diseases. It is widely used in tumor and autoimmune diseases, and monoclonal antibody has become the primary treatment for some diseases. In the field of anti-infectives, various antibody products targeting SARS-CoV-2 have recently received conditional approval or emergency authorization (Corti et al., 2021; Kaplon et al., 2022). The rapid approval of these anti-SARS-CoV-2 antibodies is a remarkable achievement and represents the fastest development of antibody therapy to date. However, antibody engineering may increase the risk of patients developing immunogenicity, including the generation of anti-drug antibodies (ADAs). ADAs can potentially result in the loss of therapeutic antibody efficacy, acceleration of clearance due to the formation of immune complexes with the therapeutic antibody, reduction in the safety and effectiveness of the drug, and even the termination of clinical trials (Carter and Rajpal, 2022). However, the antibodies studied now can reduce their immunogenicity to a certain extent after humanization. In addition, anti-fungal monoclonal antibodies are generally not used repeatedly for a long time, and most patients with invasive fungal infections have impaired immune function, so immunogenicity will not be the main factor affecting the use of anti-fungal antibodies.

Recent studies have shown that specific antibodies can provide varying degrees of protection against systemic candidiasis and mucosal candidiasis (Pellon et al., 2020). Protein mannosyltransferase (Pmt4) initiates O-mannosylation of secreted proteins, which is necessary for the formation of *Candida albicans* cell wall and its virulence. Anti-rPmt4 monoclonal antibodies may provide immune therapeutic intervention against disseminated candidiasis by modulating phagocytosis and killing activities (Wang et al., 2022). Anti-Hyr1p antibodies synergistically clear mixed biofilms of *Acinetobacter baumannii* and *Candida albicans* with antibiotics revealing a novel crosskingdom immunotherapeutic strategies that target healthcare-associated MDR infections (Uppuluri et al., 2018).

Heat shock proteins exert diverse functions in the pathogenic processes of various microorganisms (Aertsen et al., 2004; Shonhai, 2010; Verghese et al., 2012; Lamoth et al., 2016). The Ssa protein are conserved member of the Hsp70 family in yeast, exhibiting functions in heat shock protection, protein folding assistance, and transmembrane transport (Needham et al., 2015). Ssa1 is a major antigen on the cell wall of *Candida albicans* and is capable of inducing cell-mediated immune responses during host colonization by *Candida albicans* (Puri and Edgerton, 2014). Research had revealed that the ssa1Δ/Δ mutant exhibits reduced abilities in adhesion, invasion, and damage to endothelial cells and oral epithelial cells. Moreover, in murine bloodstream dissemination and oropharyngeal candidiasis, Ssa1 is indispensable for Candida albicans’ maximum virulence (Sun et al., 2010). Our findings demonstrated that cell wall-localized *C. albicans* Ssa1 could adhere to epithelial cells and mediate endocytosis, resulting in host cell damage. Additionally, our investigation revealed that the monoclonal antibody mAb 13F4, specifically directed against Ssa1, exhibited the ability to inhibit *C. albicans* adhesion and exerted effective control over candidemia *in vivo*. Therefore, we blocked *C. albicans* Ssa1 using mAb 13F4 and explored the impact on systemic infection *in vitro* and *in vivo*.

Neutralization is the most direct mechanism by which antibodies eliminate pathogens. The Fc region of antibodies can recruit innate immune cells to participate in effector functions such as opsonocytophagic, antibody-dependent cellular cytotoxicity (ADCC), or complement-dependent cytotoxicity (CDC) (Lu et al., 2018). Our study identified and characterized mAb 13F4, which has a high affinity for *C. albicans* Ssa1 (Figure 2B). We discovered that mAb 13F4 could bind to *C. albicans*, blocking its adherence to host epithelial cells (Figures 2C, 3). Since *C. albicans*-mediated adhesion to and endocytosis of host epithelial cells promotes invasive infections, in an in vitro model of host cell-Candida albicans interaction, we investigated the therapeutic effects of mAb 13F4 against infection. We discovered that mAb 13F4 can reduce the damage of Candida albicans to epithelial cells (Figure 4). Due to the ability of mAb 13F4 to simultaneously block Ssa1 and activate the immune system for opsonization, we conducted further studies using a mouse model of candidemia. The results showed that the combination of mAb 13F4 with fluconazole exhibited significant antifungal activity in mice (Figure 5). Our data suggest that mAb 13F4 is a potential therapeutic agent for treating invasive *C. albicans* infection.

To further understand mAb 13F4’s interaction with Ssa1, we constructed a homology model and found 8 amino acid contact residues in the Ssa1 nucleotide-binding domain (NBD). Hsp70s use ATP binding and hydrolysis at an NBD to regulate client polypeptide binding and release at a substrate-binding domain (SBD; Wang et al., 2021). mAb 13F4 neutralized *C. albicans* damage *in vitro* and *in vivo* and interacted with Ssa1 NBD at several amino acid residues (Figure 6). It suggests that mAb 13F4 blocked antigens by binding to these amino acids.

In conclusion, our results showed that mAb 13F4 developed against the Ssa1 protein, can inhibit *C. albicans* host cell association *in vitro*, attenuate kidney fungal burden and pathological injury *in vivo*, and improve survival. Although further research is needed to define mAb 13F4’s inhibitory mechanism, this study demonstrates that Ssa1 inhibition by mAb 13F4 can prevent *C. albicans* adherence to and “subversion” of host epithelial cells, offering a promising novel therapeutic approach for invasive fungal infections.

## Data availability statement

The original contributions presented in the study are included in the article/Supplementary material, further inquiries can be directed to the corresponding authors.

## Author contributions

X-RQ: experimental designer and executor of this research, completing data analysis, and writing and revising the first draft of the manuscript. C-RS, L-WJ, PJ, YZ, and W-TH: resources, validation, investigation, and visualization. WZ, HS, and M-MA: conceptualization, supervision, project administration, and writing—review and editing. All authors contributed to the article and approved the submitted version.

## Funding

This study was supported by the National Key Research and Development Program of China (2021YFC2300400), the Shanghai Natural Science Foundation (20ZR1459500), the National Natural Science Foundation of China (82173864), and the Shanghai Science and Technology SIKupport Program (20S11904800). The funder played no role in the study design, data collection, analysis, interpretation, or manuscript writing.

## Conflict of interest

The authors declare that the research was conducted in the absence of any commercial or financial relationships that could be construed as a potential conflict of interest.

## Publisher’s note

All claims expressed in this article are solely those of the authors and do not necessarily represent those of their affiliated organizations, or those of the publisher, the editors and the reviewers. Any product that may be evaluated in this article, or claim that may be made by its manufacturer, is not guaranteed or endorsed by the publisher.

## Footnotes

^1^https://swissmodel.expasy.org/

^2^https://zdock.umassmed.edu/
