# Supplementary figures and images for: Ssa1-targeted antibody prevents host invasion by Candida albicans
Source: Front Microbiol. 2023 Jul 25;14:1182914. doi: 10.3389/fmicb.2023.1182914 (PMC10407798; doi:10.3389/fmicb.2023.1182914)

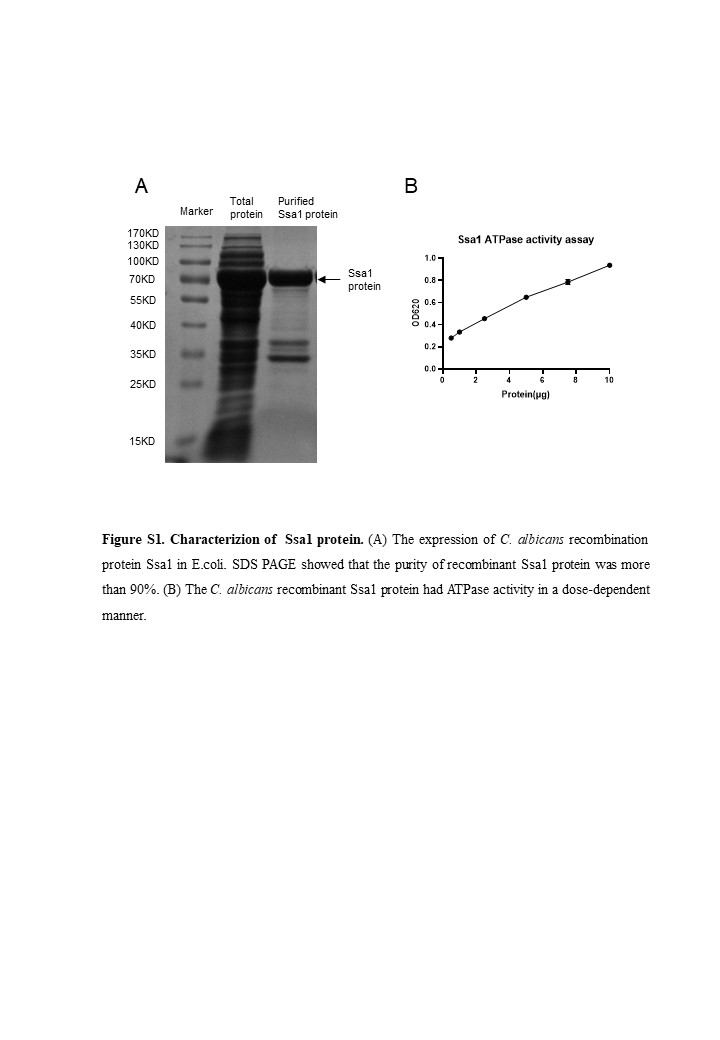

Supplement: Supplementary file 1 [file Image_1.JPEG]
